# The impact of healthy pregnancy on features of heart rate variability and pulse wave morphology derived from wrist-worn photoplethysmography

**DOI:** 10.1038/s41598-023-47980-2

**Published:** 2023-11-30

**Authors:** M. Bester, M. J. Almario Escorcia, P. Fonseca, M. Mollura, M. M. van Gilst, R. Barbieri, M. Mischi, J. O. E. H. van Laar, R. Vullings, R. Joshi

**Affiliations:** 1 Department of Electrical Engineering, Eindhoven University of Technology, 5612 AZ Eindhoven, The Netherlands; 2 Patient Care and Monitoring, Philips Research, 5656 AE Eindhoven, The Netherlands; 3 Department of Electronics, Information and Bioengineering, Politecnico di Milano, 20133 Milan, MI Italy; 4 Sleep Medicine Center Kempenhaeghe, 5591 VE Heeze, The Netherlands; 5 Department of Obstetrics and Gynecology, Máxima Medical Centrum, De Run 4600, 5504 DB Veldhoven, The Netherlands

**Keywords:** Biomedical engineering, Physiology, Time series, Autonomic nervous system, Reproductive signs and symptoms

## Abstract

Due to the association between dysfunctional maternal autonomic regulation and pregnancy complications, tracking non-invasive features of autonomic regulation derived from wrist-worn photoplethysmography (PPG) measurements may allow for the early detection of deteriorations in maternal health. However, even though a plethora of these features—specifically, features describing heart rate variability (HRV) and the morphology of the PPG waveform (morphological features)—exist in the literature, it is unclear which of these may be valuable for tracking maternal health. As an initial step towards clarity, we compute comprehensive sets of HRV and morphological features from nighttime PPG measurements. From these, using logistic regression and stepwise forward feature elimination, we identify the features that best differentiate healthy pregnant women from non-pregnant women, since these likely capture physiological adaptations necessary for sustaining healthy pregnancy. Overall, morphological features were more valuable for discriminating between pregnant and non-pregnant women than HRV features (area under the receiver operating characteristics curve of 0.825 and 0.74, respectively), with the systolic pulse wave deterioration being the most valuable single feature, followed by mean heart rate (HR). Additionally, we stratified the analysis by sleep stages and found that using features calculated only from periods of deep sleep enhanced the differences between the two groups. In conclusion, we postulate that in addition to HRV features, morphological features may also be useful in tracking maternal health and suggest specific features to be included in future research concerning maternal health.

## Introduction

During pregnancy, continuous and finely tuned physiological changes occur to maintain maternal health while supporting the growing fetus^1^. Adaptations in the maternal autonomic nervous system (ANS) are particularly important, given that the ANS regulates involuntary physiological processes such as respiration, blood pressure, and heart rate (HR), and is consequently essential to maintaining homeostasis throughout this physiologically dynamic period^2^. In comparison to healthy pregnancies, altered maternal autonomic regulation has been found in women who develop pregnancy complications such as hypertensive disorders of pregnancy (HDP) or gestational diabetes mellitus (GDM), even as early as in the first trimester^3,4^. While pregnancy complications are typically detected after the time window for clinical intervention has passed, earlier detection can improve maternal and perinatal outcomes by allowing for adequate management and treatment^5–7^.

Since dysfunctional maternal autonomic regulation has been found in women with pregnancy complications^3,4,8–11^, there is ongoing research into the potential of tracking maternal autonomic regulation to detect early deteriorations in maternal health^12–15^. Autonomic regulation can be longitudinally assessed by tracking heart rate variability (HRV) via wearable HR monitors. Longitudinal HRV tracking might be measured by photoplethysmography (PPG) recorded from wearable HR monitors such as smartwatches. PPG is an optical measure that captures blood-volume changes in the vasculature, from which HR and HRV can be derived^16^. Additionally, features describing PPG pulse wave morphology can also be determined^17,18^, here forth referred to as morphological features. While the exact physiological interpretation of morphological features is not as well-established as that of HRV, these features reflect changes in vascular tone^19^—which is autonomically regulated—and may offer additional, complementary information. Furthermore, as pregnancy necessitates vasodilation of the systematic vasculature to prevent hypertension from developing during gestation, these features might be particularly useful in capturing changes in the maternal physiology essential to a healthy pregnancy.

Numerous HRV and morphological features have been considered in the literature, and it is unclear which of these would be valuable for assessing maternal health. Research into the characteristics of the autonomic dysfunction that precedes the onset of different types of pregnancy complications is ongoing^2,9,10,20^, and consequently, it is uncertain which of these non-invasive surrogate measures of autonomic function would be best suited to identifying these impending complications. However, a reasonable starting point would be to identify the features which differ the most between healthy pregnant and healthy non-pregnant women, as these potentially reflect changes in the physiology of a healthy pregnancy and are likely to be altered in complicated pregnancies.

In this work, we compare a comprehensive set of HRV and morphological features between healthy pregnant and non-pregnant women. Comparisons of HRV between these groups have been performed by our group and others based on ECG recordings^21,22^, but none have done so using PPG measurements with relatively low sampling rates. The latter would likely be the modality used if regular tracking of autonomic activity were to be implemented as part of antenatal care^23^. Furthermore, research on the PPG waveform in pregnancy is very limited; to our knowledge, only one study has compared a limited number of morphological features between pregnant and non-pregnant women^24^.

To establish which features from the available sets of HRV and morphological features are most impacted by pregnancy, we employ a binary classification model. With the model, we can identify which features contribute the most to discriminating between these two groups. Furthermore, as we use nighttime recordings in this work, we perform a sub-analysis to explore the impact of stratifying the analysis per sleep stage. Sleep stages approximate a pseudo-controlled environment that both groups share, reducing potential environmental and circadian influences^25^ on the HRV and morphological features. Furthermore, each sleep stage is governed by a different autonomic state^26^, which could potentially enhance or elucidate differences in features between the groups that are less apparent when using data from the entire night.

## Methods

In this section, we detail the datasets used (Section “Datasets”), the preprocessing of the PPG recordings (Section “Pre-processing of the PPG data”.), and the extraction of the HRV and morphological features (Section “Feature extraction”). Next, we describe the analyses. First, we detail the binary classification model and the corresponding feature selection in Section “Logistic regression model and feature selection”. This is followed by the sleep scoring of the PPG measurements and the subsequent stratification of the classification analysis by sleep stage (Section “Stratification by sleep stages”). Finally, we describe an investigation into the effect of gestational age on the HRV and morphological features (Section “Comparison between different gestational ages”).

### Datasets

Two retrospective datasets were analyzed during this study, one containing data from healthy pregnant women and the other with data from healthy, non-pregnant women of childbearing age^27^. PPG and accelerometry measurements for both groups were acquired using the Elan sensor (Philips Electronics Nederland B.V.), a wristband that contains the Cardio and Motion Monitoring Module (CM3 Generation-3), which includes a reflective single-wavelength (green) PPG sensor and a triaxial accelerometer data^28,29^. Per the protocols of the original studies, data for the non-pregnant group were acquired at 32 Hz, while data for the pregnant group was acquired at 128 Hz. For the secondary purposes of this study, data for the pregnant group were downsampled to 32 Hz to enable comparison to the non-pregnant group. For both groups, accelerometry data were collected at 128 Hz.

For the pregnant group, as part of a volunteer study, forty-five healthy women with uncomplicated, singleton pregnancies were recruited during their second or third trimester of pregnancy. These participants were at least 18 years old, nulliparous, and had a body mass index (BMI) of between 18 and 30 kg/m^2^. Participants had no pregnancy complications or history of cardiovascular or psychiatric disease. Furthermore, participants did not use any blood pressure or sleep medication. During the study, participants were asked to wear the wristband at home for two nighttime measurement sessions approximately eight weeks apart, attaching the wristband when going to bed and removing it upon waking. PPG measurements of sufficient quality and duration to be used for sleep scoring (further described in Section “Stratification by sleep stages”) were included in the analysis; subsequently, 36 recordings are included from the first night and 30 are included from the second night. This volunteer study and its methods, which was carried out in the Netherlands in 2015, were approved by the Internal Committee of Biomedical Experiments of Philips Research, Eindhoven, the Netherlands; all participants provided written informed consent to participate in the study. All methods were performed in accordance with the Declaration of Helsinki. Participant characteristics are found in Table 1. Note that recordings from the first night are used for the comparison against non-pregnant women (Section “Logistic regression model and feature selection”), while recordings from both nights are used to assess the impact of gestational age on HRV and morphological features (Section “Comparison between different gestational ages”).

**Table 1:** Demographic information of the groups, presented as median and interquartile range.

	Pregnant group	Non-pregnant group
Number of participants	36 (first night)	36
	30 (second night)	
Age	31 (28–33) years	24 (21– 28) years
BMI (pre-pregnancy)	23.0 (20.7–25.5) kg/m^2^	23.1 (22.1–24.6) kg/m^2^
Gestational age (first night)	21 (18–23) weeks	
Gestational age (second night)	29 (26–32) weeks	
Recording length	8 h 15 min (7 h 48 min–8 h 37 min)	9 h 39 min (8 h 56 min–10 h 21 min)

*BMI* body mass index.

Data for the non-pregnant group were selected from a larger dataset of healthy volunteers recruited for a sleep study (2017 and 2018)^27^. One night of measurement was acquired per participant at a sleep clinic (Kempenhaeghe, Heeze, The Netherlands). Exclusion criteria for the data collection were indications of depression, anxiety, neurologic or psychiatric disorders, and the use of any medications, apart from birth control. Importantly, pregnancy served as an exclusion criterion. Data from all women in this group who were between the ages of 18 and 45 were available for analysis. Of these women, 36 had measurements of sufficient quality for sleep stage classification and were subsequently included. Participant characteristics are found in Table 1. The study and its methods were originally approved by the medical ethics committee of Maxima Medical Center, Veldhoven, the Netherlands (W17.128). All participants provided written informed consent to participate in the study. The use of the data for the secondary investigation presented in this paper was approved by the medical ethics committee of Sleep Medicine Center Kempenhaeghe, the Netherlands (CSG_2022_007). All methods were performed in accordance with the Declaration of Helsinki.

### Pre-processing of the PPG data

Preprocessing of the raw PPG data from the wristband was needed to facilitate feature extraction. Data were first filtered to remove information that was not physiologically relevant. Segments with motion artifacts (which often plague recordings from wrist-worn devices such as the ones used in this study) were removed. Finally, the processed PPG data were further segmented to identify fiducial points and isolate each pulse wave for feature calculation.

#### Filtering

A third-order Butterworth band-pass filter with a high-pass cutoff frequency of 0.007 Hz and a low-pass cutoff frequency of 10 Hz was applied to suppress noise. These cutoff frequencies were chosen based on examples from the literature^28,30,31^, as well as on the evaluation of the power spectral density (PSD) estimate of the raw PPG signals from the datasets (obtained with Welch’s method).

#### Removal of motion artifacts

The Signal Instability Index (SII) was calculated based on the PPG data to detect signal segments with motion artifacts. The SII is a non-parametric measure based on the probability density function of a physiological signal, calculated using kernels density estimation (KDE), employing Gaussian kernels^32^. KDE is calculated as follows:

1 $$ \widehat{{f_{h} }}\left( x \right) = \frac{1}{nh}\mathop \sum \limits_{i = 1}^{n} K\left( {\frac{{x - x_{i} }}{h}} \right), $$

where *n* is the number of equally distributed points that divide the length of the signal $x$, $K$ is the Gaussian kernel centered at the point $i$, and $h$ is the bandwidth of the Gaussian kernels. The bandwidth of the KDE is the SII, which was calculated based on 15 s epochs with a one-second sliding window^32^. Periods of the PPG signal where the SII exceeded an empirically chosen threshold of 0.8* $\sigma +\mu$ (i.e., mean of the SII plus 0.8 times the standard deviation) were noted as motion artifacts and excluded from the analysis.

#### Segmentation of PPG waveforms

**Figure 1:**
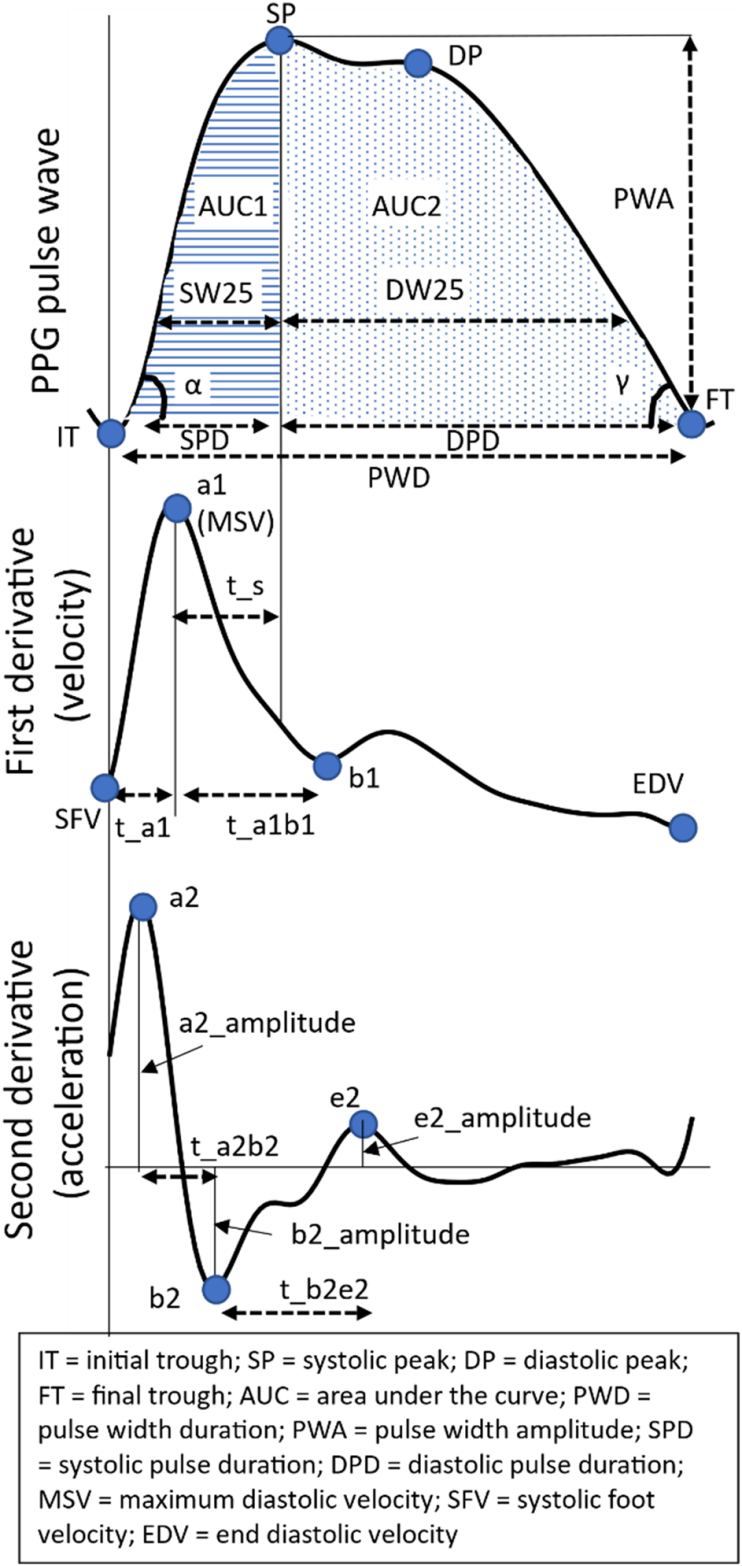
The PPG waveform and its first and second derivatives. The top waveform represents the PPG pulse wave, followed by the first and second derivatives of the waveform. Aspects of these waveforms relevant to the morphological features listed in Table 2 are indicated in the figures.

Hereafter, the PPG signal was segmented using the pulse segmentation method developed by Elgendi et al.^18^, as implemented in the NeuroKit2 package in Python^33^. Thereafter, a further refinement step was performed. To ensure the systolic peak (SP) was not misdetected (see Fig. 1, Section “Morphological features”), the detected peak was checked against point e2 from the second derivative of the PPG pulse wave (Fig. 1), which corresponds to the notch between the SP and diastolic peak (DP). If the detected peak occurred after this reference point, the SP was redefined as the peak between the initial trough (IT in Fig. 1) and point e.

### Feature extraction

Features based on the variability between heartbeats (HRV features) and the morphology of the pulse wave (morphological features) were calculated. These features are computed based on five-minute measurement segments, as is further elaborated upon in Section “Data analysis and statistics”. Considering the large number of features calculated (n = 67), these are briefly described or illustrated in the following sections. In all cases, references are added, which provide more detailed information.

#### Heart rate variability

HRV is a family of features that describe the fluctuation in the duration of the interbeat intervals (IBIs) over time, resulting from the complex and non-linear oscillations of the heart. This fluctuation is regulated by the dynamic relationship between the two ANS branches, namely the parasympathetic and sympathetic nervous systems^34,35^. Specific selections of HRV features were calculated with time-domain, frequency-domain, non-linear, phase rectified signal averaging (PRSA), and heart rate fragmentation (HRF) analyses^35–38^. For all the methods, the IBIs were computed based on the distance between two consecutive pulse wave troughs. To further eliminate erratic or incorrectly detected IBIs, those that varied more than 20% from the preceding IBI or were not between 0.3 and 2.4 s in duration were removed^30^. For methods requiring a continuous signal, i.e., frequency-domain features and some non-linear features such as sample entropy, the IBIs were interpolated using an on-time approach detailed elsewhere^39^, in which the timestamps of the missing heartbeats are found with quadratic interpolation.

##### *Time domain features*^35,40–42^

- Mean HR: The mean IBIs per segment, converted to beats per minute (bpm).
- SDNN: Standard deviation of normal-to-normal (NN) IBIs. SDNN is related to the total variability of the HR and is impacted by both the sympathetic and parasympathetic nervous systems.
- RMSSD: Root mean square of the time differences between successive normal heartbeats. RMSSD estimates the beat-to-beat variance of HR mediated by the vagus nerve, which is the main component of the PNS.
- pNN50: Percentage of contiguous IBIs which differ with more than 50 ms. This feature measures the parasympathetic modulation of IBIs^41^.
- Kurtosis: The kurtosis of the spread of the IBIs.
- Skewness: The skewness of the spread of the IBIs.

##### Frequency-domain features

The power spectral density and the subsequent features estimations were performed with the Fast Fourier Transform (FFT) algorithm, based on the Welch method and using pyHRV, an open-source Python toolbox for the computation of HRV parameters^38,43^. IBI series are divided into shorter, overlapping segments (5 min in length, 50% overlap) during computation, and the mean of the values computed per segment is taken as the result for the corresponding IBI series. The following features were calculated^35,40,41^:

- Very-low-frequency (VLF) power: Absolute power of frequency band between 0 and 0.04 Hz. Information about the physiological mechanisms of which the activity is reflected in this band is uncertain, but it has been linked mainly to PNS activity.
- Low-frequency (LF) power: Absolute power of frequency band between 0.04 and 0.15 Hz, mainly reflecting baroreceptor activity.
- High-frequency (HF) power: Absolute power of frequency band between 0.15 and 0.4 Hz. HF power reflects parasympathetic activity, with respiration having a major contribution.
- Normalized LF: LF power normalized by the sum of LF and HF powers.
- Normalized HF: HF power normalized by the sum of LF and HF powers.
- LF/HF: This ratio is considered a measure of sympathovagal balance.

##### Non-linear features

Non-linear features aim to capture the regularity or complexity of the IBIs^35,41^. For this analysis, the pyHRV toolbox was also used. The Poincaré plot was first determined, in which a scatter plot is obtained by plotting the IBIs against their precursor, to then fit an ellipse. From this ellipse, the width (SD1) and length (SD2) are determined, which capture short-term and long-term variability, respectively^44^. Furthermore, the SD1/SD2 is calculated, which represents the relationship between short- and long-term variability and is correlated with the LF/HF ratio^35,45^. Finally, the area of the ellipse (S) is determined, giving a measure of total HRV.

Additionally, the self-similarity of the IBIs over time was analyzed using detrended fluctuation analysis (DFA). Rather than being fully predictable or completely random, patterns within the HR signal are expected to repeat over different timescales. To capture these correlations, the short-term (4 to 16 beats) fractal scaling exponent from DFA is calculated, namely, ${\alpha }_{1}$^46^. Additionally, sample entropy (SampEn) is calculated to assess the complexity of the IBIs. SampEn determines the conditional probability that two epochs that are similar within a tolerance *r* for a window length *m*, will remain similar when including the next data point (i.e., the next IBI)^47,48^. The parameters *m* and *r* were set to 2 and 0.2 times the standard deviation of the IBIs^47^. Lower SampEn indicates a more regular and predictable time series.

##### PRSA features

Quasi-periodicities may exist within the HR which are obscured by noise. PRSA is a signal analysis method that can detect such quasi-periodicities in physiological signals to assess system dynamics, regardless of the noise that is typically present. Briefly, this method compresses the signal into a shorter, averaged waveform that captures the relevant quasi-periodicities while discarding non-stationarities, artifacts, and noise by synchronizing the phase of the periodic components^36,49^.

The method comprises three steps. First, the phases of interest are identified, referred to as the anchor points (APs). Here, there are two sets, HR accelerations, and HR decelerations. Thereafter, a signal segment is identified around each AP. Next, these segments are aligned by their AP and averaged, resulting in a waveform that captures the behavior of the signal relative to the AP. If there is no periodicity linked to the AP, this averaging would result in a flatline. However, if such a periodicity exists, the averaging should result in a waveform with oscillations.

This waveform is described with several features. Consider the case where HR decelerations are the APs. Deceleration capacity (DC) is calculated to capture the magnitude of the response in the waveform to the AP by summing the value of the two points preceding the AP, the value at the AP, before diving this sum by four. Furthermore, the immediate deceleration response (IDR) is computed as the difference between maximum and minimum values of the waveform in the five data points preceding the AP and the five thereafter, including the AP. Correspondingly, the slope of this deceleration response (SDR) is determined. Finally, the average deceleration response (ADR) is estimated as the differences between the mean of the 50 values preceding the APs and the mean of the 50 values thereafter, including the AP. Similarly, these features are also calculated for the case where HR accelerations are the APs: acceleration capacity (AC), immediate acceleration response (IAR), slope of the acceleration response (SAR), and average acceleration response (AAR)^36,50^.

##### HRF features

This method is used to discern whether the short-term dynamics of the HRV are being vagally or non-vagally mediated. If the short-term variation is smooth, it is likely vagally mediated. Conversely, if this variation is jagged, or fragmented, it likely results from a breakdown in physiological control over HR rather than healthy autonomic modulation. The features which were developed to capture this HRF are the following: PIP (percentage inflection points); PAS (percentage alternating segments); PSS (percentage short segments); and IALS (inverse of accelerating or decelerating long segments). Increases in these features indicate increased fragmentation in HR^37^.

#### Morphological features

The PPG pulse wave mainly reflects blood flow dynamics through the vascular bed^17^. The rising edge of the pulse reflects the systolic phase of the heartbeat (i.e., between IT and SP in Fig. 1), and the diastolic phase is reflected in the falling edge (i.e., between SP and the final trough (FT) in Fig. 1). Morphological features were calculated from the literature^30,51,52^. In addition, features describing angles, slopes, and velocity were added. Only the fully segmented pulses were considered for feature extraction, i.e., pulses for which the IT, the SP, and the FT were all detected, as shown in Fig. 1. The PPG waveform as well as the waveforms resulting from its first and second derivatives were used to compute several features; these waveforms and their relative characteristics are detailed in Fig. 1. Note that the first and second derivatives reflect the velocity and acceleration of the PPG waveform, respectively^53^. The morphological features, listed in Table 2, are clustered by amplitudes, time differences, areas under the curve (AUCs), velocity and acceleration, ratios, slopes, and angles. It should be noted that most of these features do not yet have a clear physiological interpretation but rather attempt to capture the characteristics of the pulse as fully as possible.

**Table 2:** Description of the morphological features.

Features		Explanation
Amplitude	PWA	Pulse width amplitude, i.e., the difference between SP and IT
	b2_amplitude	The absolute value of the amplitude of the deepest trough of the second derivative signal (b2)
Time differences	PWD	Pulse width duration; time interval between IT and FT
	SPD	Systolic phase duration; time interval between IT and SP
	DPD	Diastolic phase duration; time interval between SP and FT
	t_a1	Time interval between IT and a1 on the first derivative signal
	t_a1b1	Time interval between the a1 and the first valley of the first derivative signal (b1)
	t_a2b2	Time interval between points a2 and b2 on the second derivative signal
	t_b2e2	Time interval between points b2 and e2 on the second derivative signal
AUC	AUC_total	AUC of the full pulse wave, i.e., between IT and FT
	AUC1	AUC of systolic phase, i.e., between IT and SP
	AUC2	AUC of diastolic phase, i.e., between SP and FT
Velocity and acceleration	mean(V)	Mean velocity, i.e., mean of the first derivative signal
	IDR(V)	Interdecile range of velocity, i.e., interdecile range of the first derivative signal
	Mean (Acc)	Mean of the second derivative signal
	MSV	Max systolic velocity; a1 on the first derivative
	SFV	Systolic foot velocity, i.e., value of the point on the first derivative signal corresponding to IT of the pulse wave
Ratio	DW10/SW10	The ratio of systolic width to diastolic width at 10% of the pulse wave amplitude; similar features are calculated at 25, 50, and 60%
	t_s/PWD	The ratio between the time interval between a1 and SP (i.e., t_s), and the pulse width duration (PWD), which is the time interval between IT and FT
	t_a1/PWD	The ratio of the time interval between the IT and a1 (i.e., t_a1) to PWD
	t_a1b1/PWD	The ratio of t_a1b1 to PWD
	t_a2b2/PWD	The ratio of t_a2b2 to PWD
	t_b2e2/PWD	The ratio of t_b2e2 to PWD
	b2/a2	The ratio of b2_amplitude to a2_amplitude, found on the second derivative signal
	e2/a2	The ratio of e2_amplitude to a2_amplitude, found on the second derivative signal
	SPD/PWD	The ratio of SPD to PWD
	SP/SPD	The ratio of the value of SP to SPD
	Pulsatility index	(Max systolic velocity (i.e., a1 on the first derivative) − end diastolic velocity (i.e., EDV on the first derivative)/(mean of the first derivative)
Slope	slope_IT_SP	The slope of line that connects IT and SP
	slope_SP_FT	The slope of line that connects SP and FT
Angle	α	The angle of the slope between IT and SP
	γ	The angle of the slope between SP and FT

The fiducial points on the PPG pulse wave discussed in the table can be found in Fig. 1. Note that in Fig. 1, DW25 and SW25 can be found, which represent the diastolic and systolic widths at 25% of the amplitude. Where features such as DW are mentioned in the table, these are similar to DW25, but at 10% instead of 25%.

### Data analysis and statistics

The features described in Section “Feature extraction” are calculated based on 5-min segments of non-overlapping PPG data. Morphological features are obtained for every pulse and then averaged over the full segment. Segments are discarded if 20% of the data in the segment was removed during motion artifact correction (Section “Removal of motion artifacts”), or if 20% of the IBIs are deemed unreliable as defined in Section “Heart rate variability”. Once all the features are calculated, these are used for the classification model discussed in Section “Logistic regression model and feature selection” below. Furthermore, we compare these features between the pregnant and non-pregnant groups using the Mann–Whitney-U test for statistical significance, along with Cohen’s *d* for effect size, reported with 95% confidence interval^54^.

#### Logistic regression model and feature selection

In this work, we develop three sets of models based on only data extracted from wrist-worn PPG measurements. The first set uses only HRV features, the second only morphological features, and the third a combination of HRV and morphological features. Before developing the models, we first obtained the feature rankings for each feature set. The feature importance was estimated based on a stepwise forward elimination process, which was performed for each of the seven runs of a seven-fold cross-validation, which was empirically chosen. Features were ranked according to their importance for each iteration and ultimately the ten most popular features across all iterations were selected to be used for each of the feature sets. Note that we do not consider PWD (pulse width duration) for the morphological feature set, as PWD is analogous to mean HR, which forms part of the HRV features.

Thereafter, we developed logistic regression models based on the features identified for each of the feature sets, respectively, to classify women as either pregnant or non-pregnant. The classification is first performed using only the most important feature identified for each feature set. Thereafter, the second most important feature for each set is incorporated and the classification is repeated. The rest of the ten features for each feature set are likewise introduced into the model one by one. The predictive strength of the classifiers was evaluated by calculating the area under the receiver operating characteristic curve (AUROC) from the left-out folds of a sevenfold cross-validation, repeated seven times to provide an average estimate of the AUROC along with its standard deviation to provide a measure of dispersion. Furthermore, considering that multiple measurements are available per participant, the classification was also repeated with measurements stratified per participant.

The AUROC was calculated with two aims in mind; first, to assess the ability of the classifiers to discriminate between pregnant and non-pregnant women, and second, to capture the additional contribution of each of the features incorporated into the models. Note that the data for the pregnant group comprises recordings from the first night of the pregnancy dataset.

#### Stratification by sleep stages

As a sub-analysis, we repeat the analysis detailed in Section “Logistic regression model and feature selection” using features calculated only from data from a specific sleep stage. Sleep stages are determined for both datasets using a published, automated algorithm that scores sleep based on PPG data and accompanying accelerometer data^55^. Sleep scoring is done per 30-s epoch, classifying data as light sleep (N1/N2), deep sleep (N3), rapid eye movement (REM), and Wake^55^. Note that only the data from the identified sleep stages are used and Wake data are discarded for this sub-analysis. Again, the segments used are non-overlapping PPG segments of five minutes, as described in Section “Data analysis and statistics”, each of which now contain data from only one sleep stage.

#### Comparison between different gestational ages

As a further sub-analysis, we compare the features identified in Section “Logistic regression model and feature selection” between the first and second night of recordings of the pregnant group to assess whether the features capture the changes of progressing gestation.

#### Investigation on impact of age

As there is a median difference of six years between the ages of the groups (Table 1), we investigate if there is a trend in the most important features (Section “Logistic regression model and feature selection”) relative to age. To this end, we perform a linear regression for each of the important features, using age as the independent variable. If multiple 5-min segments were available per participant, these were averaged to provide one feature value.

## Results

### Descriptive statistics of HRV and morphological features

Differences in the HRV and morphological features, as calculated based on five-minute segments from the full recordings, are tabulated in Supplementary Materials in Tables S1 and S2, respectively. The vast majority of features differed significantly between the groups, with the largest effect sizes found for the following features, reported with 95% confidence interval: SPD (*d* = 1.03 (0.98–1.09)), mean HR (0.93 (0.86–1.01)), t_a1 (*d* = 0.82 (0.74–0.89)), DW10/SW10 (d = 0.72 (0.66–0.78)), SPD/PWD (d = 0.66 (0.60–0.72)), RMSSD (*d* = 0.65 (0.59–0.72)) and SD1 (*d* = 0.65 (0.59–0.73)). The latter two features relate to parasympathetic modulation, while those before all relate to the cardiac cycle to some extent.

**Figure 2:**
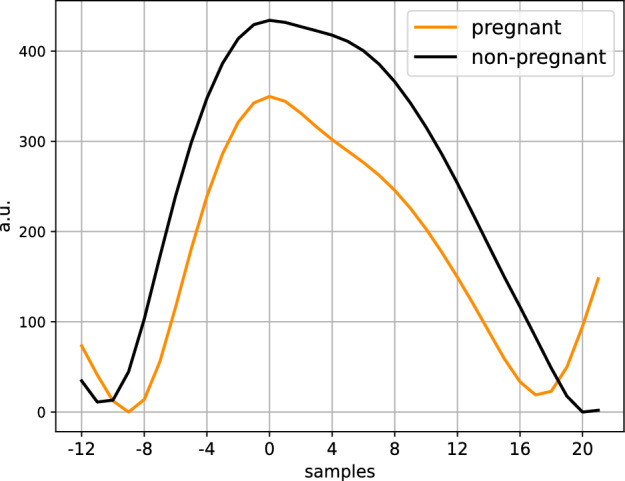
Ensemble plots of the PPG waveform for a pregnant and non-pregnant participant to visualize the differences between the groups. The resulting waveform is computed using the full night’s recording and the waveforms are centered (sample = 0) on the peaks.

To offer some context on how the PPG morphology differs between the pregnant and non-pregnant group, Fig. 2 shows an ensemble plot of the PPG waveforms for a pregnant and non-pregnant participant, computed using the full night’s measurement. On this plot differences such as the smaller SPD and lower PWA can clearly be observed in the pregnant participant as compared to the non-pregnant participant.

### Feature importance

In Table 3, the features which were identified to be most valuable in discriminating between pregnant and non-pregnant women are listed for each feature set. Note that for HRV, mean HR and PSS were consistently the most and second most important features in each iteration; the same is true for SPD and t_ab for both the morphological feature set, as well as the combined feature set. Additionally, IALS was the third most important feature for the combination of features in each iteration.

**Table 3:** The most important features for discriminating between pregnant and non-pregnant women, as chosen by stepwise forward elimination, for each of the feature sets as well as the combination of the two feature sets.

Importance	HRV	Morphology	HRV + Morphology
1	Mean HR	SPD	SPD
2	PSS	t_a2b2	t_a2b2
3	SAR	IDR(V)	IALS
4	SDR	FSV	FSV
5	S (Poincaré)	AUC2	b2_amplitude
6	PIP	b2_amplitude	slope_IT_SP
7	AC	SP/SPD	PWA
8	IAR	t_s/PWD	S (Poincaré)
9	IDR	PWA	SP/SPD
10	SD2	slope_IT_SP	AUC1

### Logistic regression model

The number of five-minute data segments available for the classification model is listed in Table 4, both considering the entire recording as well as per sleep stage. Furthermore, the number of participants for which data were available in each stratified analysis is also listed. Note that these numbers are lower than the total number of participants as in some cases participants did not have five-minute measurements available which were continuously in the relevant sleep stage and of sufficient quality, as defined in Section “Data analysis and statistics”. Considering the sleep stages, the highest number of segments are available for light sleep (N1/N2), substantially less for deep sleep (N3), and the least for REM sleep.

**Table 4:** Number of measurements available for the classification model based on the entire night’s recording, as well as only N1/N2, N3, or REM, respectively.

	Pregnant group		Non-pregnant group	
	No. of segments	No. of participants	No. of segments	No. of participants
Full night	1629	36	1407	36
N1/N2 (light sleep)	903	33	788	32
N3 (deep sleep)	200	29	288	30
REM	152	23	172	27

**Figure 3:**
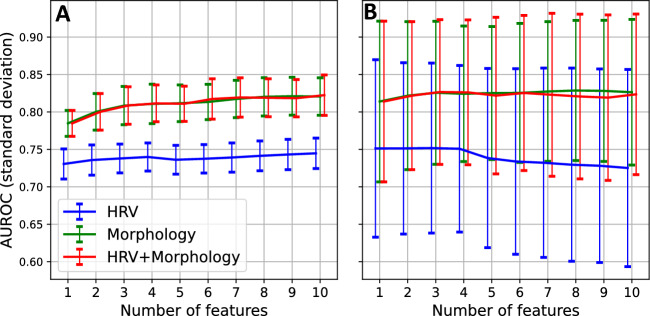
Classification of pregnant and non-pregnant women based on full night recordings. (**A**) The AUROC scores, with standard deviation, for the models based on HRV features only (blue), morphological features only (green), and a combination of the two sets (red), plotted against the number of features used. In this case, all the segments are considered unique, disregarding the subject. (**B**) AUROC scores, with standard deviation, for classification based on HRV features only (blue), morphological features only (green), and a combination of the two sets (blue), stratified by participant, i.e., when the measurements of each participant are grouped, plotted against the number of features used. The features correspond to those listed in Table 3. For example, when three features are used, the first three features listed in Table 3 have been used.

Figure 3 shows the AUROC scores (with standard deviation), for the classification based on only HRV features, only morphological features, and a combination of the two feature sets. The classification is first performed using one feature, thereafter two features, etc. until ten features are reached. The features used are the ones detailed in Table 3. To illustrate, consider the HRV features: first, only mean HR is used for the classification; next, mean HR and PSS are both used; thereafter, mean HR, PSS, and SAR are used; etc.

The results in the left panel (A) reflect the performance of the model when all measurement segments are treated as unique, while in the right panel (B), the classification was repeated, now with measurement segments stratified per participant. The performance of the models, as measured with the AUROC, remains comparable between panels A and B. However, when stratifying per participant the performance variance increases substantially. In both cases, notice that the classifiers using only PPG or a combination of PPG and HRV perform comparably well while using only HRV results in the poorer performance. Comparable variance in the performance is seen for each feature set. Furthermore, for the HRV feature set, the addition of features to mean HR offers only slight improvements to the performance (Fig. 3A) or none at all (Fig. 3B), at least not within the ten best-ranked features. For the morphological feature set or the combination feature set, incremental improvements are seen in performance when adding additional features to SPD.

### Sleep stage stratification

**Figure 4:**
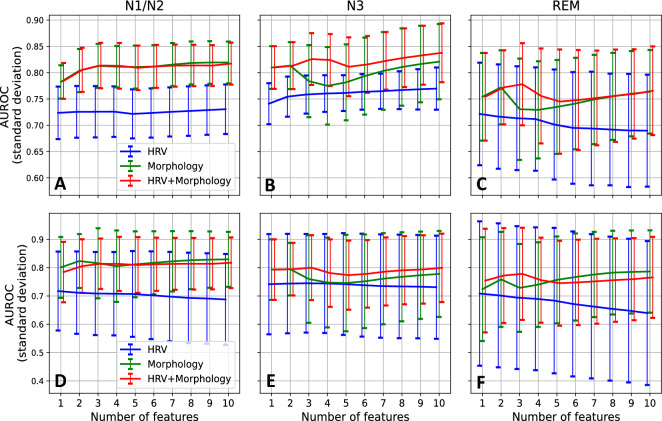
Classification of pregnant and non-pregnant women based on data from individual sleep stages. AUROC scores, with standard deviation, for the models using only data from (**A**) the N1/N2 (light sleep), (**B**) N3 (deep sleep), or (**C**) REM sleep stages. For each, the results of three models are presented, based on HRV features only (blue), morphological features only (green), and a combination of the two sets (red), respectively. In (**D**), (**E**), and (**F**), the AUROC scores with standard deviation are again presented per sleep stage but now stratified on participant level.

The classification process in the previous section is repeated, now based only on data from each scored sleep stage (Fig. 4), namely N1/N2, N3, and REM. Considering the performance of the models at ten features, the model performs best when data from N3 are used (Fig. 4B), although the improvement compared to that of N1/N2 is small (Fig. 4A). The performance of the latter closely resembles the performance based on data from the entire night (Fig. 3A), likely in part due to N1/N2 being the most prevalent sleep stage. The classifier performs worst when data from the REM stage is used (Fig. 4C). In panels D to F, the analyses presented in panels A to C are repeated, this time stratified on participant level. The performance is comparable to that seen in panels A to C but with substantially increased variance.

Furthermore, notice the classification based on only HRV features for the N3 stage (Fig. 3B); even at only one feature, the classifier outperforms the ones based on data from N1/N2 or REM. Moreover, in N3 we observe that incorporating further HRV features systematically improves the model’s performance.

### The impact of gestational age on HRV and morphological features

The features which were identified as important were further compared between the recordings of the first and second nights of the pregnant group. Differences in these HRV and morphological features are tabulated in Table S3 in Supplementary material. Concerning the HRV features, there were significant differences between the groups for all features. Mean HR further increased in later pregnancy, while most features that were reduced compared to non-pregnant women, such as RMSSD and IALS, decreased further. For the morphological features, fewer features significantly differed between the gestational age groups, although the area under the PPG curve (as assessed with AUC1 and AUC2), as well as the PWA (pulse wave amplitude), continued to decrease. Noticeably, SPD, which is the most important feature for discriminating between pregnant and non-pregnant women (Table 3) does not change with progressing pregnancy, at least not within the eight weeks between the first and second night of measurements.

### The impact of age on the top ten features

Regression models were performed to assess the relationship between age and the ten most important features (Table 3). The two most important features, SPD and t_a2b2, were not significantly correlated with age. The two HRV features—IALS and SPD—showed a significant relationship with age (*p* < 0.05). Of the remaining six features, which are all morphological features, only AUC1 was significantly correlated with age.

## Discussion

Various wearable monitors are available to track cardiac activity, of which the most popular are wrist-worn PPG monitors. As a healthy pregnancy necessitates continuous changes in maternal autonomic regulation, tracking HRV is considered to be a potential tool for assessing maternal health^12,13,56–58^. In addition to HRV features, morphological features—which describe the autonomically regulated morphology of the pulse wave—can also be extracted from PPG measurements^17^. While many HRV and morphological features exist in the literature, typically used in other application areas, it is unclear which of these would be valuable in assessing maternal health. As a foundational step towards identifying potentially useful features, we investigated which of these are most important in discriminating between healthy pregnant and non-pregnant women.

Based on a stepwise forward elimination process, we find that SPD (systolic phase duration) and mean HR are, individually, the most important features for discriminating between these two groups (Table 3), with an AUROC of 0.78 and 0.73, respectively (Fig. 3A). It stands to reason that the difference in SPD between pregnant and non-pregnant women is largely driven by the increased mean HR observed in the pregnant group. However, if we consider the differences in the DPD (diastolic phase duration) and SPD/PWD, i.e., the ratio of the SPD to the pulse wave duration (in Table S2), we can observe that the relative decrease in SPD during pregnancy is larger than the decrease in DPD. Therefore, SPD likely outperforms mean HR because it reflects not only the autonomic changes that accompany gestation but also, to some extent, the cardiovascular adaptions which occur during pregnancy. The overall performance of the models (Figs. 2 and 3) further supports this. In all cases, using only morphological features results in an improved performance when compared to using only HRV features. Furthermore, if we consider the performance of the combination of HRV and morphological features, we see that this improves considerably on the performance of only HRV but is generally comparable to using only morphological features.

Consequently, we suggest that the longitudinal tracking of maternal HRV with wearable PPG monitors would benefit from also incorporating morphological features. Specifically, SPD, t_a2b2, IDR(V) (i.e., the interdecile range of the first derivative), the FSV (foot systolic velocity), and b2_amplitude appear to be particularly valuable. However, it is important to note that while HRV features are reasonably explainable^35^, substantial future work is needed to determine the physiology underpinning morphological features, especially during pregnancy. Yet, considering our results, along with the fact that the data for determining these features can be easily acquired, we believe that morphological features are of additional value and should be included in the assessment of the maternal condition. Moreover, morphological features may in the future be particularly useful in identifying pregnancy complications such as HDP, since these are associated with cardiovascular dysfunction as well as autonomic dysfunction. Furthermore, preeclampsia (a type of HDP) is characterized by endothelial damage to the systematic vasculature, which may be detectable with morphological features^57,59,60^.

Considering the HRV features, we notice that for the model based only on these features, the addition of further HRV features to the mean HR does not seem to improve the model performance (Figs. 2A, 3A, and C). Yet, research indicates that healthy pregnant women have increased sympathetic and decreased parasympathetic activity compared to healthy controls^2,22,61,62^. While this autonomic imbalance does contribute to the increased HR that we see in pregnant women^63,64^, it is doubtful that HRV features offer no additional physiological information. Potentially, it is the low sampling rate (32 Hz) of PPG that offers inadequate precision to fully capture differences in HRV. Additionally, mean HR is likely more robust to arousals than HRV, as the latter is a sensitive metric. This theory is further supported by the fact that the value of the HRV features is more evident during deep sleep (N3, Fig. 4B), which is the sleep stage with the least amount of arousals^65^.

Half of the HRV features identified as important (Table 3) for discriminating between pregnant and non-pregnant women are those of the PRSA analysis, suggesting that this category of features should be incorporated in future assessments of maternal health. These features are linked to autonomic responsiveness, and potentially reflect the damped physiological responsiveness which is observed during pregnancy^66,67^. Moreover, PRSA features are particularly robust against noise^36,49^, which is advantageous when assessing PPG data collected in free-living conditions. Additionally, HRF features also play an important role, with PSS and IALS being the second most important feature in the HRV feature set and the third most important feature in the combined feature set, respectively (Table 3). The purpose of HRF features is to capture variability in the IBIs which is not vagally regulated^37^; therefore, these may add unique value since they capture information that is not reflected in the majority of HRV features, which capture some aspect of vagal or sympathetic activity^35^. Subsequently, in addition to PRSA features, HRF features may also be valuable in tracking maternal health.

Furthermore, the results for the classification using HRV features calculated from deep sleep data (N3) are interesting (Fig. 4B). Here the classification performance is comparable to the other sleep stages when using mean HR only, but thereafter it increases incrementally to the best performance at ten features. During deep sleep, movements are minimal; therefore, measurements from this sleep stage may be of higher quality than other stages, allowing for more accurate HRV analysis. Additionally, considering that pregnant women have reduced parasympathetic activity compared to non-pregnant women and that deep sleep is a state of parasympathetic dominance^26^, the autonomic differences between these two groups may be heightened during N3 sleep, making HRV more impactful in discriminating between the two groups. Furthermore, HRV features are likely more robust during N3 since autonomic arousals—which introduce brief bursts of sympathetic activity—are at a minimum during deep sleep^65^. This hypothesis is further strengthened when noting the performance of the classifier using only features calculated from REM data (Fig. 4C). During REM sleep, sympathetic influence is increased^26^. While healthy pregnant women also have increased sympathetic activity compared to non-pregnant women, REM is characterized by regular shifts in autonomic balance^26^, potentially obscuring physiological differences between the groups which are more prominent during deep sleep.

Consequently, we postulate that when tracking non-invasive indices of maternal autonomic regulation to assess maternal health, it would be beneficial to focus on data from the N3 sleep stage. Doing so might elucidate differences in these features which are not apparent when performing assessments based on the entire night’s recording or based on a specific timepoint, e.g., using data recorded daily at 05:00 h. This sleep stage can act as a pseudo-controlled environment in free-living conditions that repeats nightly, with minimum motion artifacts and a stable autonomic state. This would allow for tracking the progression of these features over time with reduced influence from environmental and circadian factors. Additionally, considering again that the ultimate goal would be to detect pregnancy complications early in pregnancy, using N3 data would exploit prior knowledge of differences between healthy and complicated pregnancies, namely that complicated pregnancies have reduced parasympathetic activity compared to healthy pregnancies^10,11^.

The study presented here has some limitations, primarily the relatively low sampling rate of the PPG measurements (32 Hz). The necessary sampling rate to determine HRV from PPG measurements is widely debated; however, feature accuracy is improved when measurements have been collected at rest, as is the case in this work^68^. When we compare our HRV results (Table S1, Supplementary material) to prior work based on ECG measurements^21^, we observe similar trends, i.e., features linked to parasympathetic activity (e.g., RMSDD and HF), PRSA features, and HRF features are generally lower in the pregnant group, while mean HR is increased. Still, in future work, PPG measurements of a higher sampling rate should be used; alternatively, features that are the most susceptible to errors due to low sampling rates, such as frequency domain features, should be excluded. Furthermore, while we find in this work that HRV features perform poorer than morphological features in discriminating between pregnant and non-pregnant women, HRV features may perform better at higher sampling frequencies. However, performance may also similarly improve in this case for the morphology features.

The reliability of morphological features at different sampling rates has been less extensively researched, although one study has shown that several features are reliable even at a sampling rate of 30 Hz^69^. Still, despite the low sampling rate, we observe robust differences between our groups (Supplementary material). A further unknown in this analysis is the positioning of the participants wrist. While it is known that the wrist position may influence the amplitude of the PPG waveform, it is unlikely that the effect of this would be larger than the effect of pregnancy. Moreover, due to the longitudinal nature of the measurements and the fact that participants are asleep, it would not be possible to control for wrist positioning.

Another limitation is that there is an age difference of six years between our groups. While age is known to impact HRV, the impact of this small difference is unlikely to be larger than the impact of pregnancy itself^70^. From our sub analysis (Sections “Investigation on impact of age” and “The impact of age on the top ten features”), we do indeed see that the two HRV features which form part of the ten most important features (Table 3) are correlated with age. However, the two features which are most valuable for discriminating between pregnant and non-pregnant women (SPD and t_a2b2), as well as the majority of the rest of the morphological features, are not significantly influenced by this factor. Still, a prospective, age-matched study is necessary to confirm our results.

Finally, an algorithm that scores sleep stages based on PPG data was used for sleep scoring in this analysis, rather than sleep scoring performed by a technician based on polysomnography data. Using this automated sleep scorer, which has been shown to have reasonable accuracy^55^, allows for comparable sleep scoring in both datasets. Furthermore, using an algorithm that scores sleep stages based only on PPG data and accompanying accelerometer data would allow for sleep staging information to be incorporated in longitudinal assessments of maternal health in free-living conditions, as suggested earlier in this discussion.

To conclude, we have demonstrated that in addition to differences in HRV, there are also significant differences in morphological features between healthy pregnant and non-pregnant women. SPD (systolic pulse duration) and mean HR are the most important features for discriminating between these two groups, based on PPG measurements at a relatively low sampling rate (32 Hz). Moreover, morphological features were overall more valuable for discriminating between the groups than HRV features. We suggest that morphological features may in the future be valuable for tracking maternal health. Furthermore, when using HRV to assess maternal health, PRSA and HRF should be included in the analyses.

### Supplementary Information

Supplementary Tables.

## Data Availability

Data is not publicly available but may be made available at reasonable request to the corresponding author.
