# Supplementary material for: The impact of healthy pregnancy on features of heart rate variability and pulse wave morphology derived from wrist-worn photoplethysmography
Source: Sci Rep. 2023 Nov 30;13:21100. doi: 10.1038/s41598-023-47980-2 (PMC10689737; doi:10.1038/s41598-023-47980-2)
Supplement: Supplementary file 1 — Supplementary Tables. [file 41598_2023_47980_MOESM1_ESM.pdf]

## Supplementary information

**Table S1**: HRV features, presented as median and interquartile range, along with significance and effect size of differences between pregnant and non-pregnant women (*p*-value and *d*-value)

| Features | | Pregnant group | Non-pregnant group | *p*-value | *d*-value (95% confidence interval) |
|---|---|---|---|---|---|
| *Time domain* | Mean HR | 65.43 (60.91 - 69.43) | 59.19 (54.13 - 63.84) | **< 0.0001** | 0.93 (0.86 - 1.01) |
| | SDNN | 57.92 (43.67 - 76.75) | 71.08 (50.8 - 92.5) | **< 0.0001** | 0.28 (0.21 - 0.38) |
| | RMSSD | 51.87 (40.57 - 62.96) | 66.83 (46.09 - 88.22) | **< 0.0001** | 0.65 (0.59 - 0.72) |
| | pNN50 | 34.97 (22.12 - 44.92) | 45.66 (28.65 - 59.03) | **< 0.0001** | 0.56 (0.49 - 0.64) |
| *Frequency domain* | VLF | 740.06 (346.13 - 1536.69) | 955.4 (449.91 - 2038.09) | **< 0.0001** | 0.24 (0.18 - 0.3) |
| | LF | 900.79 (400.04 - 2049.59) | 1350.22 (587.1 - 2656.8) | **< 0.0001** | 0.28 (0.21 - 0.34) |
| | HF | 1167.39 (591.57 - 2237.11) | 1970.65 (808.53 - 3947.78) | **< 0.0001** | 0.42 (0.35 - 0.48) |
| | Normalized LF | 0.43 (0.29 - 0.58) | 0.4 (0.29 - 0.54) | **0.0022** | 0.13 (0.06 - 0.2) |
| | Normalized HF | 0.57 (0.42 - 0.71) | 0.6 (0.46 - 0.71) | **0.0022** | 0.13 (0.06 - 0.2) |
| | LF/HF | 0.76 (0.41 - 1.38) | 0.68 (0.4 - 1.18) | **0.0022** | 0.19 (0.13 - 0.26) |
| *Non-linear* | SD1 | 36.68 (28.69 - 44.49) | 47.19 (32.59 - 62.11) | **< 0.0001** | 0.65 (0.59 - 0.73) |
| | SD2 | 67.68 (49.62 - 90.77) | 82.44 (58.12 - 107.18) | **< 0.0001** | 0.39 (0.32 - 0.46) |
| | SD1/SD2 | 0.55 (0.43 - 0.68) | 0.6 (0.47 - 0.74) | **< 0.0001** | 0.25 (0.18 - 0.32) |
| | S | 7741.15 (4820.81 - 12246.44) | 12057.77 (6467.05 - 20484.69) | **< 0.0001** | 0.57 (0.51 - 0.64) |
| | $\alpha_1$ | 0.86 (0.7 - 1.04) | 0.83 (0.68 - 0.97) | **< 0.0001** | 0.18 (0.11 - 0.25) |
| | SampEn | 1.49 (1.26 - 1.68) | 1.65 (1.37 - 1.95) | **< 0.0001** | 0.54 (0.46 - 0.61) |
| *PRSA* | DC | 21.06 (15.62 - 27.55) | 24.0 (17.89 - 32.23) | **< 0.0001** | 0.29 (0.21 - 0.36) |
| | AC | -21.57 (-28.71 - -16.33) | -25.61 (-36.01 - -19.33) | **< 0.0001** | 0.41 (0.33 - 0.48) |
| | IDR | 53.43 (44.41 - 65.35) | 65.84 (46.88 - 87.01) | **< 0.0001** | 0.49 (0.42 - 0.56) |
| | IAR | 55.21 (44.2 - 68.29) | 66.4 (49.91 - 88.87) | **< 0.0001** | 0.48 (0.41 - 0.55) |
| | SDR | 40.24 (27.14 - 51.68) | 42.2 (21.05 - 59.85) | 0.081 | 0.05 (-0.02 - 0.12) |
| | SAR | -40.86 (-53.58 - -29.35) | -46.88 (-68.24 - -33.98) | **< 0.0001** | 0.32 (0.25 - 0.4) |
| | ADR | 1.37 (-2.02 - 5.41) | 1.27 (-2.83 - 6.08) | 0.2638 | 0.08 (0.01 - 0.15) |
| | AAR | -2.05 (-5.89 - 1.49) | -2.36 (-6.98 - 1.74) | 0.0849 | 0.07 (-0.0 - 0.14) |
| *HRF* | PIP | 74.79 (66.55 - 82.51) | 71.02 (62.35 - 78.8) | **< 0.0001** | 0.28 (0.21 - 0.35) |
| | PAS | 0.38 (0.34 - 0.41) | 0.41 (0.37 - 0.45) | **< 0.0001** | 0.62 (0.55 - 0.7) |
| | PSS | 54.68 (47.1 - 67.8) | 63.97 (54.35 - 79.9) | **< 0.0001** | 0.64 (0.57 - 0.71) |
| | IALS | 50.59 (46.45 - 54.81) | 51.52 (47.42 - 55.86) | **< 0.0001** | 0.22 (0.15 - 0.29) |
| *Spread* | Kurtosis | 0.54 (-0.14 - 2.19) | 0.21 (-0.33 - 1.49) | **< 0.0001** | 0.06 (-0.01 - 0.13) |
| | Skewness | -0.08 (-0.68 - 0.48) | -0.11 (-0.67 - 0.27) | 0.1183 | 0.09 (-0.04 - 0.2) |
| Rejected IBIs (%) | | 2 (0 - 4) | 2 (0 - 6) | **0.0001** | 0.22 (0.15 - 0.29) |

**Table S2**: Morphological features, presented as median and interquartile range, along with significance and effect size of differences between pregnant and non-pregnant women (*p*-value and *d*-value). The last metric, i.e., Missing data, refers to the percentage of the PPG segment which was disregarded due to motion artifacts (Section 2.2.2).

| Features | | Pregnant group | Non-pregnant group | *p*-value | *d*-value (95% confidence interval) |
|---|---|---|---|---|---|
| *Ampli -tude* | PWA | 292.67 (208.0 - 413.53) | 329.43 (196.81 - 524.88) | **< 0.0001** | 0.2 (-0.13 - 0.27 ) |
| | b_amplitude | 15.97 (10.3 - 25.14) | 12.89 (7.21 - 20.43) | **< 0.0001** | 0.39 (0.32 - 0.45) |
| *Time differences* | SPD | 0.26 (0.25 - 0.28) | 0.3 (0.28 - 0.33) | **< 0.0001** | 1.03 (0.98 - 1.09) |
| | DPD | 0.66 (0.61 - 0.72) | 0.69 (0.64 - 0.76) | **< 0.0001** | 0.41 (0.34 - 0.48) |
| | t_a1 | 0.08 (0.07 - 0.09) | 0.09 (0.08 - 0.09) | **< 0.0001** | 0.82 (0.74 - 0.89) |
| | t_a1b1 | 0.25 (0.24 - 0.27) | 0.27 (0.25 - 0.31) | **< 0.0001** | 0.65 (0.59 - 0.71) |
| | t_a2b2 | 0.34 (0.32 - 0.36) | 0.36 (0.34 - 0.39) | **< 0.0001** | 0.51 (0.44 - 0.59) |
| | t_b2e2 | 0.24 (0.22 - 0.25) | 0.23 (0.2 - 0.25) | **< 0.0001** | 0.18 (0.11 - 0.24) |
| *AUC* | AUC Tot | 72.52 (48.27 - 100.98) | 88.2 (55.24 - 140.78) | **< 0.0001** | 0.39 (0.33 - 0.46) |
| | AUC1 | 21.7 (14.46 - 30.18) | 28.74 (17.78 - 42.56) | **< 0.0001** | 0.47 (0.4 - 0.54) |
| | AUC2 | 46.74 (30.34 - 66.46) | 55.34 (34.81 - 89.9) | **< 0.0001** | 0.37 (0.31 - 0.44) |
| *Velocity/ acceleration* | mean(V) | 0.02 (-0.02 - 0.13) | -0.0 (-0.08 - 0.04) | **< 0.0001** | 0.2 (0.13 - 0.33) |
| | IDR(V) | 2.2 (1.47 - 3.3) | 2.82 (1.79 - 4.13) | **< 0.0001** | 0.14 (0.02 - 0.3) |
| | mean(Acc) | -0.02 (-0.05 - 0.0) | -0.01 (-0.04 - 0.0) | 0.9334 | 0.0 (-0.07 - 0.07) |
| | MSV | 63.5 (43.56 - 93.11) | 59.79 (34.74 - 95.22) | **0.001** | 0.12 (0.06 - 0.19) |
| | SFV | 14.45 (9.51 - 21.53) | 13.43 (7.54 - 21.1) | **< 0.0001** | 0.17 (0.11 - 0.24) |
| *Ratio* | DW10/SW10 | 2.77 (2.53 - 3.13) | 2.56 (2.29 - 2.84) | **< 0.0001** | 0.72 (0.66 - 0.78) |
| | DW25/SW25 | 2.86 (2.62 - 3.22) | 2.64 (2.37 - 2.93) | **< 0.0001** | 0.7 (0.64 - 0.76) |
| | DW50/SW50 | 2.77 (2.55 - 3.13) | 2.64 (2.4 - 2.91) | **< 0.0001** | 0.54 (0.48 - 0.61) |
| | DW66/SW66 | 2.62 (2.4 - 2.92) | 2.56 (2.34 - 2.84) | **< 0.0001** | 0.22 (0.15 - 0.29) |
| | t_s/PWD | 0.01 (0.01 - 0.01) | 0.01 (0.01 - 0.01) | **< 0.0001** | 0.63 (0.57 - 0.69) |
| | t_a1/PWD | 0.08 (0.07 - 0.09) | 0.09 (0.08 - 0.09) | **< 0.0001** | 0.31 (0.24 - 0.39) |
| | t_a1b1/PWD | 0.27 (0.25 - 0.28) | 0.28 (0.25 - 0.3) | **< 0.0001** | 0.5 (0.44 - 0.56) |
| | t_a2b2/PWD | 0.35 (0.33 - 0.38) | 0.36 (0.33 - 0.39) | 0.0038 | 0.01 (-0.06 - 0.08) |
| | t_b2e2/PWD | 0.24 (0.22 - 0.26) | 0.23 (0.2 - 0.25) | **< 0.0001** | 0.14 (0.07 - 0.22) |
| | b2/a2 | 7.68 (-5.55 - 22.72) | 6.28 (-2.98 - 18.22) | 0.2547 | 0.04 (-0.02 - 0.08) |
| | e2/a2 | 1.62 (-1.18 - 4.83) | 1.23 (-0.54 - 3.76) | 0.1158 | 0.03 (-0.04 - 0.08) |
| | SPD/PWD | 0.29 (0.27 - 0.31) | 0.3 (0.28 - 0.33) | **< 0.0001** | 0.66 (0.6 - 0.72) |
| | SP/SPD | 34.57 (24.47 - 49.53) | 34.32 (18.89 - 54.36) | 0.0738 | 0.03 (-0.04 - 0.1) |
| | Pulsatility index | 10.84 (-65.42 - 90.45) | 3.03 (-59.8 - 73.06) | 0.063 | 0.06 (-0.01 - 0.11) |
| *Slope* | slope_IT_SP | 1106.12 (783.0 - 1584.88) | 1098.29 (604.48 - 1739.57) | 0.0738 | 0.03 (-0.04 - 0.1) |
| | slope_SP_FT | -434.82 (-629.44 - -309.37) | -495.13 (-761.95 - -286.64) | **0.0022** | 0.1 (0.03 - 0.18) |
| *An-gle* | α | 1.57 (1.57 - 1.57) | 1.57 (1.57 - 1.57) | 0.0821 | 0.27 (0.15 - 0.39) |
| | γ | 1.57 (1.57 - 1.57) | 1.57 (1.57 - 1.57) | 0.1855 | 0.07 (-0.05 - 0.19) |
| Missing data (%) | | 7 (2 - 13) | 6 (2 - 12) | **0.0001** | 0.13 (0.06 - 0.2) |

**Table S3**: Statistical significance and effect sizes (*p*-value and *d*-value) of differences in selected HRV and morphological features (Table 3) between N1 and N2 measurements for the pregnant group. Results are presented as median with interquartile range.

| **Features** | | **Pregnant group (night 1)** | **Pregnant group (night 2)** | ***p*-value** | ***d*-value (95% confidence interval)** |
|---|---|---|---|---|---|
| **HRV features** | Mean HR | 65.43 (60.91 - 69.43) | 67.77 (63.34 - 72.64) | **< 0.0001** | 0.39 (0.3 - 0.47) |
| | PSS | 54.68 (47.1 - 67.8) | 51.51 (45.23 - 60.63) | **< 0.0001** | 0.3 (0.22 - 0.38) |
| | SAR | -40.86 (-53.58 - -29.35) | -39.29 (-47.55 - -27.47) | **< 0.0001** | 0.15 (0.07 - 0.23) |
| | SDR | 40.24 (27.14 - 51.68) | 38.63 (25.33 - 47.07) | **< 0.0001** | 0.14 (0.06 - 0.22) |
| | S (Poincaré) | 7741.15 (4820.81 - 12246.44) | 6469.14 (4280.16 - 9796.16) | **< 0.0001** | 0.34 (0.26 - 0.42) |
| | PIP | 74.79 (66.55 - 82.51) | 75.85 (67.78 - 83.5) | **0.045** | 0.11 (0.02 - 0.19) |
| | AC | -21.57 (-28.71 - -16.33) | -20.71 (-27.35 - -15.68) | **0.0224** | 0.16 (0.08 - 0.24) |
| | IAR | 55.21 (44.2 - 68.29) | 50.55 (42.46 - 62.94) | **< 0.0001** | 0.29 (0.21 - 0.36) |
| | IDR | 53.43 (44.41 - 65.35) | 49.37 (42.44 - 60.59) | **< 0.0001** | 0.26 (0.18 - 0.34) |
| | SD2 | 67.68 (49.62 - 90.77) | 61.48 (48.03 - 80.38) | **< 0.0001** | 0.22 (0.14 - 0.3) |
| | IALS | 0.38 (0.34 - 0.41) | 0.37 (0.33 - 0.39) | **< 0.0001** | 0.22 (0.14 - 0.3) |
| **Morphological features** | SPD | 0.26 (0.25 - 0.28) | 0.26 (0.24 - 0.28) | 0.1962 | 0.09 (0.01 - 0.18) |
| | t_a2b2 | 0.34 (0.32 - 0.36) | 0.34 (0.31 - 0.36) | **0.0174** | 0.12 (0.04 - 0.21) |
| | IDR(V) | 2.2 (1.47 - 3.3) | 2.09 (1.42 - 3.07) | 0.0571 | 0.1 (0.04 - 0.17) |
| | FSV | 14.45 (9.51 - 21.53) | 12.89 (8.59 - 19.34) | **0.0002** | 0.18 (0.11 - 0.26) |
| | AUC2 | 46.74 (30.34 - 66.46) | 39.81 (28.84 - 55.57) | **< 0.0001** | 0.24 (0.15 - 0.32) |
| | b_amplitude | 15.97 (10.3 - 25.14) | 15.84 (10.11 - 23.48) | 0.1504 | 0.1 (0.02 - 0.17) |
| | SP/SPD | 34.57 (24.47 - 49.53) | 34.34 (23.66 - 47.71) | 0.2834 | 0.09 (0.01 - 0.17) |
| | t_s/PWD | 0.01 (0.01 - 0.01) | 0.01 (0.01 - 0.01) | 0.938 | 0.06 (0.03 - 0.14) |
| | PWA | 292.67 (208.0 - 413.53) | 278.1 (199.45 - 388.11) | **0.025** | 0.15 (0.07 - 0.23) |
| | slope_IT_SP | 1106.12 (783.0 - 1584.88) | 1098.8 (757.23 - 1526.8) | 0.2834 | 0.09 (0.01 - 0.17) |
| | AUC1 | 21.7 (14.46 - 30.18) | 19.59 (14.27 - 27.06) | **0.0009** | 0.2 (0.12 - 0.27) |
